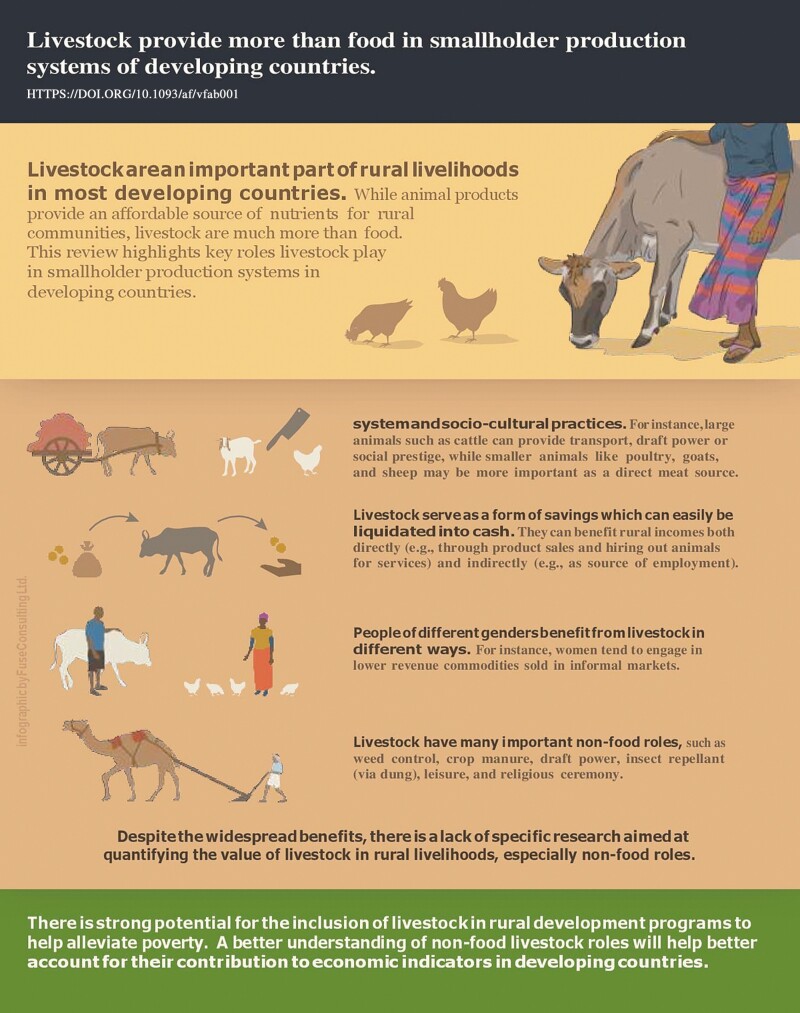# Livestock provide more than food in smallholder production systems of developing countries

**DOI:** 10.1093/af/vfab024

**Published:** 2021-05-17